# Flame Suppression in Lithium‐Ion Batteries During Thermal Runaway Through Solvent Design Considering the Volatility–Flammability Relationship

**DOI:** 10.1002/advs.77307

**Published:** 2026-08-19

**Authors:** Chi‐Yeong Hong, Joon Ha Chang, Ho Yeon Jang, Jooeun Byun, Chae Rim Lee, Jeongmin Kim, Oh B. Chae, Youngjin Kim, Jun Ho Song, Chihyun Hwang, Seoin Back, Ki Jae Kim, Hyun‐seung Kim

**Affiliations:** ^1^ Advanced Batteries Research Center Korea Electronics Technology Institute Seongnam‐si Republic of Korea; ^2^ Department of Energy Science Sungkyunkwan University Suwon Republic of Korea; ^3^ SKKU Institute of Energy Science and Technology (SIEST) Sungkyunkwan University Suwon Republic of Korea; ^4^ Department of Battery Science and Engineering Sungkyunkwan University Suwon Republic of Korea; ^5^ Department of Materials Science and Engineering Korea National University of Transportation Chungju Republic of Korea; ^6^ Department of Chemical and Biomolecular Engineering Institute of Emergent Materials Sogang University Seoul Republic of Korea; ^7^ Department of Future Energy Engineering Sungkyunkwan University Suwon Republic of Korea; ^8^ School of Chemical Biological and Battery Engineering Gachon University Seongnam‐si Republic of Korea; ^9^ Department of Battery Convergence Engineering Kangwon National University Chuncheon‐si Republic of Korea; ^10^ KU‐KIST Graduate School of Converging Science and Technology Korea University Seoul Republic of Korea; ^11^ Department of Integrated Energy Engineering Korea University Seoul Republic of Korea; ^12^ Department of Energy Sungkyunkwan University Suwon Republic of Korea

**Keywords:** nonflammable electrolyte, safety, solvent, thermal propagation, thermal runaway

## Abstract

An asymmetric sulfonate‐based linear solvent, 2,2,2‐trifluoromethyl mesylate (FMS), is designed and used as the primary solvent in liquid electrolytes to enhance the thermal safety of lithium‐ion batteries (LIBs). The highly polar FMS molecule, composed of trifluoroethyl and methyl groups asymmetrically bound to a sulfonate core, significantly suppresses solvent vaporization and shows a significantly higher flash point than the conventional LIB solvent dimethyl carbonate. Low volatility contributes to the non‐flammability of the FMS electrolyte. Upon electrochemical cycling, the FMS‐based electrolyte facilitates the formation of thermally stable SO*_x_*‐containing passivation films on both graphite and LiNi_0.8_Co_0.1_Mn_0.1_O_2_ (NCM811) electrodes. These robust interfacial films effectively suppress side reactions, reduce self‐discharge‐induced heat accumulation, and improve the overall thermal stability of the system under abusive conditions. Furthermore, the interfacial reinforcement and non‐flammability of FMS effectively suppress the thermal propagation‐induced flame hazard potential of LIBs. Without reducing the battery cycle life, the FMS electrolyte improves individual cell safety while effectively suppressing thermal propagation within stacked batteries. Additionally, the electrolyte formulation proposed in this study includes only fluoroethylene carbonate and LiPF_6_, which are both compatible with commercial LIB systems, thereby exhibiting high potential practicability in real systems.

## Graphical abstract

An asymmetric sulfonate solvent forms thermally stable interphases and demonstrates non‐flammability, suppressing heat accumulation for safer, high‐performance lithium‐ion batteries.

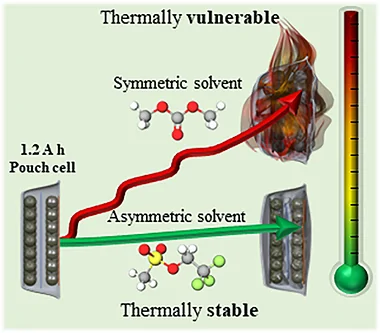

## 1 Introduction

Lithium‐ion batteries (LIBs) are widely applied in high‐energy systems, such as electric vehicles and energy storage systems [1, 2, 3, 4, 5, 6, 7, 8, 9, 10, 11]; consequently, mitigating the thermal failure of battery packs is critical for preventing flame hazards and ensuring safety [12, 13, 14, 15]. The thermal failure of LIBs is generally induced by external abuse and the incorporation of inherent defects during production; thermal runaway is typically induced by abusive conditions, resulting in the failure of the battery management system. This failure generally increases the temperature of loaded batteries [13, 14, 15, 16, 17, 18, 19]; thus, the thermal stability of LIBs should be improved to ensure safety. In general, heat accumulation in LIBs is induced by the rapid self‐discharge of stored energy from active materials, which leads to Joule heating and the chemical degradation of the system [17, 20, 21, 22, 23, 24]. Interfacial side reactions at elevated temperatures, the most prominent heat‐accumulating reactions associated with the thermal runaway of LIBs [17, 21, 22], lead to electrolyte vaporization, which increases the pressure in sealed LIBs, resulting in flame formation upon exposure to abnormal conditions. Hence, a two‐step strategy is crucial for mitigating the thermal runaway of LIBs; each stage, from initial heat accumulation to flame propagation, requires a distinct and targeted suppression mechanism. The first stage involves decreasing the interfacial side reactions on the negative and positive electrode surfaces to decrease the heat accumulation in batteries [22, 25, 26, 27, 28, 29]. A robust solid electrolyte interphase (SEI) and surface film, passivation films that reduce electron leakage during high‐temperature exposure, preventing self‐discharge‐induced heat accumulation, can be used in this stage. The second stage involves suppressing electrolyte‐solvent vaporization, preventing a heat accumulation‐induced temperature increment. The flash point of solvent molecules significantly increases with decreasing vapor pressure; thus, a less vaporizing electrolyte is crucial for improving the safety of LIBs. The non‐flammability of electrolytes is generally ensured by substituting a linear carbonate solvent with ionic liquids [30, 31, 32], perfluorinated solvents [33, 34], phosphate‐based additives [35, 36], and highly viscous solvents. Although the introduction of these components enhances the safety of the LIB system, it significantly limits the wettability and compatibility of the electrolyte at the electrode and separator components, adversely affecting the cycle performance of the system [14, 30, 33, 34, 35, 36]. Furthermore, to improve the thermal stability of electrolytes, imide‐type lithium salts are generally used in nonflammable electrolytes [17, 31]. However, imide‐type electrolytes lead to aluminum corrosion at high voltages [37, 38, 39, 40]. Because the positive electrode current collector in LIBs typically comprises aluminum, the practical application of this electrolyte system is limited compared with that of LiPF_6_‐based electrolytes.

An asymmetric sulfonate‐based linear solvent was used as the main linear solvent in the liquid electrolyte to enhance the thermal stability of LIBs in this study. To ensure non‐flammability, an asymmetric molecular design was applied to increase the flash point of the electrolyte. 2,2,2‐Trifluoromethyl mesylate (FMS) contains trifluoromethyl and methyl groups on each side of a sulfonate moiety, thereby exhibiting a highly polarized solvent structure. Hence, compared with the conventional dimethyl carbonate (DMC)‐based electrolytes used in LIBs, which typically comprise linear solvents, the vaporization of FMS is significantly slower owing to strong molecular interactions. Consequently, the flash point of FMS can be increased beyond that of DMC. Furthermore, a robust and chemically stable SO*_x_*‐based passivation film can be deposited on the negative and positive electrodes by the application of a linear solvent containing a sulfonate moiety. The passivation film with high thermal stability used in this study minimized charged electrode/electrolyte side reactions, resulting in minimal heat accumulation in the LIB. Furthermore, as the newly designed electrolyte comprises only fluoroethylene carbonate (FEC) and a LiPF_6_ salt, both common components of commercial LIBs, it exhibits high potential practicability. Additionally, FMS can be directly blended with DMC, demonstrating its practical compatibility with existing battery systems.

## 2 Results and Discussion

Figure 1 shows the working mechanism and physicochemical characterization results of the FMS and DMC electrolyte‐containing cells. Figure 1a shows schematics of the working mechanism of the FMS electrolyte, which is largely composed of asymmetric solvents that reduce the overall solvent vaporization at high temperatures. Owing to this phenomenon, lesser fueling is required in LIBs containing the FMS electrolyte than those containing the DMC electrolyte. Moreover, the low vaporization of FMS at high temperatures increases its flash point (110°C) significantly beyond that of the typical linear solvent DMC (18°C). Thus, substituting DMC with FMS resulted in a significant reduction in the flammability of the linear solvent used in this study. The FMS electrolyte formed a thermally stable SO*_x_*‐based passivation film on the positive and negative electrode surfaces, mitigating heat accumulation from film decomposition and the subsequent self‐discharge of active materials. In addition, the FMS electrolyte was less vaporized at high temperatures than DMC, leading to delayed thermal runaway and low risk of potential ignition in the LIB.

**FIGURE 1:**
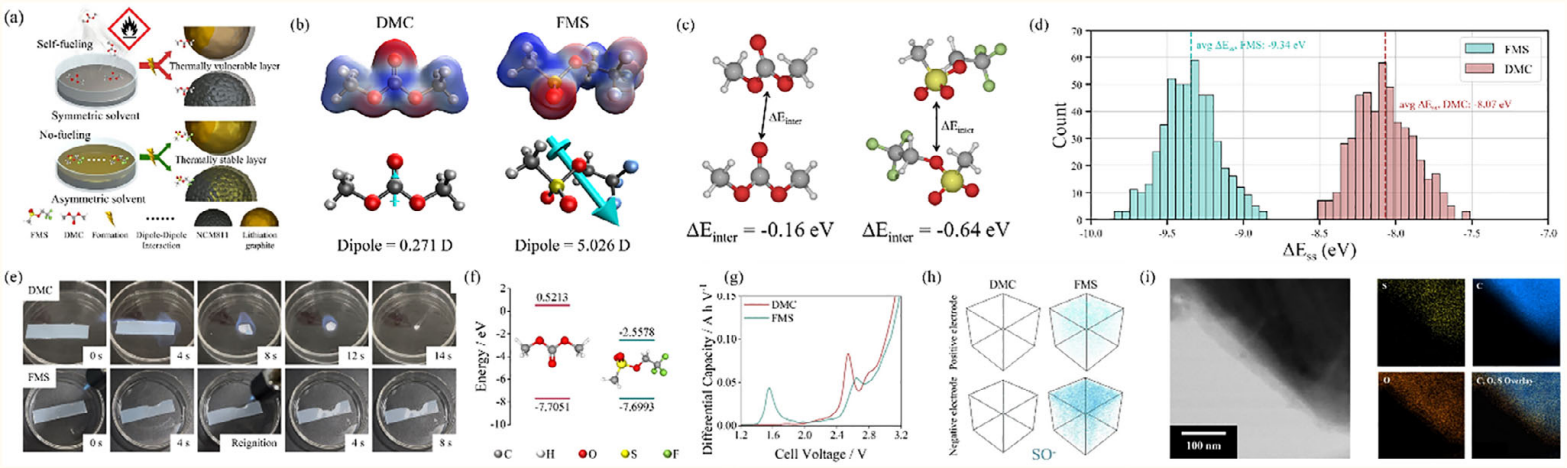
(a) Schematics of the working mechanism of the FMS electrolyte, (b) dipole moment and ESP map of DMC and FMS solvents, (c) solvent–solvent interaction energies (∆E_inter_) of DMC and FMS, (d) interaction energy distribution (∆E_SS_) between the primary solvation cluster and the surrounding electrolyte, with atomic configurations generated from AIMD simulations, (e) open‐cup flammability test images for DMC and FMS, (f) frontier orbital energy levels of the solvents DMC and FMS, (g) initial differential capacity plots of DMC and FMS electrolytes in graphite/NCM811 1.2 A h batteries, (h) ToF‐SIMS three‐dimensional maps of the positive and negative electrodes with DMC and FMS electrolytes (after formation), (i) *cryo*‐TEM image and corresponding EDS map for a graphite electrode with the FMS electrolyte.

Figure 1b,c presents the DFT‐calculated intrinsic physical properties. The dipole moments and electrostatic potential (ESP) maps of the DMC solvents differ significantly from those of an asymmetrically polarized solvent (FMS) structure (Figure 1b). The dipole moment of DMC is 0.271 D, whereas that of the FMS solvent is 5.026 D, approximately 18 times higher. This large difference in dipole moment leads to markedly different solvent–solvent interaction energies. While DMC exhibits a weak interaction energy of −0.16 eV, FMS shows a much stronger interaction energy of −0.64 eV. Consequently, the strong intermolecular interactions in FMS suppress its vaporization, as a higher energy input is required to overcome the cohesive forces between solvent molecules. To further analyze the solvation behavior, the solvent binding energy within the solvation structure (∆E*_b,i_*) and interaction energy between the primary solvation structures and the surrounding electrolyte (∆E_SS_) were calculated using ab initio molecular dynamics (AIMD) simulations (Figure 1d and Figure S1). The AIMD results indicate that FMS has an average energy barrier 1.27 eV higher than that of DMC for releasing a solvation cluster from the electrolyte. Moreover, the initial and final structures from the AIMD simulations, along with energy profiles over 20 ps are shown in Figure S2. The ∆E*_b,i_* values indicate that FMS binds more strongly to the Li^+^ ion than DMC does. These stronger binding energies and higher energy barriers suggest that the self‐fueling (autonomous release) of the FMS solvent is significantly restricted at elevated temperatures.

Figure 1e shows the self‐extinction test (SET) results for separators soaked in DMC and FMS. In the DMC‐soaked polyethylene (PE) separator, a flame is observed with subsequent thermal shrinkage, consistent with the ignition behavior of the low‐flash‐point solvent DMC. In contrast, no flame is observed in the FMS‐soaked PE separator after the SET test, implying a SET time of 0 s owing to the high flash point of FMS. Only the fire‐contacted PE‐soaked separator undergoes thermal shrinkage on separator re‐ignition, implying that the FMS solvent can be used as a nonflammable electrolyte. These results are fully consistent with the DFT and AIMD predictions, which demonstrated that the asymmetric molecular structure of FMS enhances its dipole moment and strengthens solvent–solvent interactions, thereby elevating the flash point and suppressing volatilization. The SET results thus provide direct experimental validation of the computationally predicted nonflammable character of FMS.

Figure S3 shows the contact angles of the DMC and FMS electrolytes with the LiNi_0.8_Co_0.1_Mn_0.1_O_2_ (NCM811) electrode. Although nonflammable electrolytes lack wettability at the electrodes, the FMS electrolyte exhibits a contact angle similar to that of the DMC electrolyte, indicating that the wettability of the FMS electrolyte is just sufficient to operate the LIB system.

Figure 1f shows the frontier orbital energy levels of DMC and FMS. The lowest unoccupied molecular orbital (LUMO) energy level of DMC is significantly higher than that of FMS, indicating FMS‐solvent‐driven SEI film formation on graphite, and the highest occupied molecular orbital (HOMO) energy level of FMS is lower than that of DMC, indicating surface film formation on NCM materials. The LUMO–HOMO energy levels shown in Figure 1 indicate passivation‐film modification by FMS, resulting in the formation of a thermally stable interphase.

Figure 1g shows the initial differential capacity plots of graphite/NCM811 cells with the DMC and FMS electrolytes. In the plot of the system containing the FMS electrolyte, a peak originating from FMS‐induced film formation is observed along with a peak corresponding to FEC, which shows low intensity owing to SEI film formation. In contrast, the plot of the system containing the DMC electrolyte contains no peaks at low cell voltages, with a clear FEC‐reduction peak at 2.5 V [1]. Hence, film modification is verified by differential capacity analysis during formation.

The three‐dimensional time‐of‐flight secondary ion mass spectroscopy (ToF‐SIMS) map in Figure 1h indicates a significant SO^−^ anion fractal at both the positive and negative electrode surfaces, implying sulfoxide‐based film modification in FMS. Figures S4 and S5 show the x‐ray photoelectron spectroscopy (XPS) spectra of the negative and positive electrodes cycled in the DMC and FMS electrolytes, respectively. A thin organic SEI film is formed on the graphite electrode cycled in DMC, while a thick SEI film with an Li_2_SO_3_ component is formed on the electrode cycled in FMS [41, 42]. In addition, Figure S6 shows the XPS depth profile of negative electrodes with respective electrolytes. As the etching time increases, the S 2p and O 1s concentrations increase, suggesting that the SO*_x_*‐based SEI film is formed near the graphite surface. In both systems, an SO*_x_*‐based surface film is deposited on the NCM‐material positive electrode [41, 42]; the lattice oxygen peaks from the NCM material are less intense in the FMS electrolyte than in the DMC electrolyte, implying the deposition of a denser surface film in FMS [2, 43, 44].

Figure 1i shows cryogenic transmission electron microscopy (*cryo*‐TEM) images and the corresponding energy‐dispersive X‐ray spectroscopy (EDS) map of the SEI film formed on the graphite electrode in FMS. Unlike the typical oxygen‐based SEI component, the S‐based SEI film can occupy the near‐graphite surface because FMS decomposes earlier than FEC. This result is consistent with what is expected from the XPS depth profiles. Figure S7 shows EDS spectra for corresponding EDS maps. The EDS map from the *cryo*‐TEM measurement shows an intense sulfur signal near the graphite surface, implying that the graphite surface is well protected by layer‐by‐layer SEI film formation. These results confirm that the directly attached SO*_x_*‐based SEI film formed in FMS effectively passivates graphite electrodes.

A 60°C storage test using Li/graphite half‐cells with DMC and FMS at SOC 0 was used to analyze and compare the thermal stability of the SEI films deposited on graphite electrodes in both systems (Figure S8a). The SOC 0 state is suitable for thermal storage testing because the marginal self‐discharge of the graphite electrode leads to a significant increase in the open‐circuit voltage (OCV) and the absence of SEI reformation with electrolyte exposure in this state [27, 45, 46]. The time‐dependent OCV values measured in this test indicate a large voltage increment in DMC. Interestingly, stable OCV values are observed in FMS. An increase in OCV values indicates the self‐discharge of the graphite electrode. Therefore, the results of this test indicate that the failure of the SEI film is significantly mitigated in the FMS electrolyte. Figure S8b shows the Li‐ion binding energies of the SEI film components formed in FMS and DMC. The formation of a thermally soluble SEI film leads to the self‐discharge of the graphite electrode; consequently, a higher Li‐ion binding energy, which improves the thermal stability of the SEI film, is preferred. The calculated binding energy for Li_2_SO_3_ is higher than that for lithium ethyl dicarbonate or lithium methyl carbonate, both typical SEI components [46, 47, 48, 49, 50, 51]. Therefore, a reduction in the thermal solubility of the SEI film is crucial for increasing the thermal stability of the graphite electrode at high temperatures in FMS.

Figure 2 compares the thermal reactions of graphite, NCM, and 1.2 A h LIBs in systems containing FMS and DMC. Figure 2a shows the differential scanning calorimetry (DSC) results of fully lithiated graphite electrodes soaked in FMS and DMC. The DSC profile of graphite electrodes soaked in DMC indicates significant exothermic reactions due to SEI decomposition at 115.1°C. These reactions involve the decomposition of less‐stable organic SEI components, such as lithium alkyl carbonates, while PF_5_ generated at elevated temperatures acts as a strong Lewis acid that attacks oxygen‐containing functional groups in the SEI, thereby accelerating its degradation. This is followed by a graphite–electrolyte reaction at 237.5°C [16, 18, 19]. Additionally, DMC vaporizes at 50°C, indicating a high possibility of flame propagation with conventional carbonate electrolytes. This exothermic behavior implies that release from graphite electrodes is a critical thermal failure mode in these LIBs. In contrast, the FMS electrolyte significantly reduces the heat flow caused by SEI decomposition, indicating effective graphite‐surface passivation by the Li*_x_* SO*_y_*‐based SEI film formed in the system. Consequently, compared with the DMC system, the peak temperature of the exothermic reaction in FMS is shifted upward by 21.2°C; moreover, the total heat released from SEI decomposition is reduced by 85.4%, suggesting that the introduction of the FMS electrolyte decreases heat accumulation caused by SEI film failure on the graphite electrode. Furthermore, FMS exhibits a higher vaporization temperature than DMC, thereby reducing the flame hazard potential of the system owing to reduced organic vapor formation.

**FIGURE 2:**
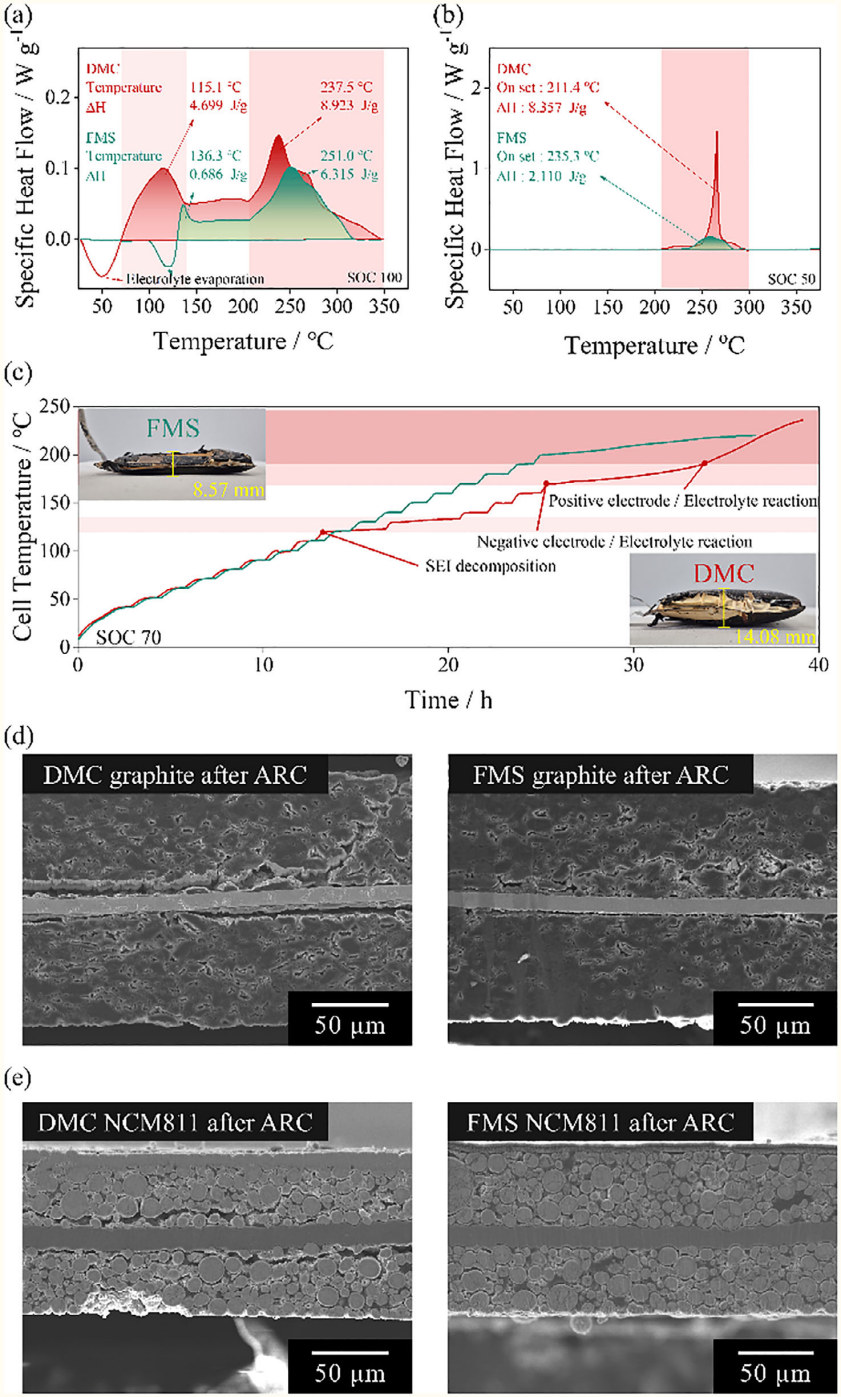
DSC plots of (a) graphite and (b) NCM811 electrodes soaked in FMS and DMC. (c) ARC profiles of 1.2 A h graphite/NCM811 pouch cells with DMC and FMS, ex situ cross‐sectional SEM image of the (d) graphite and (e) NCM811 electrodes in DMC‐ and FMS‐injected pouch cells after ARC testing.

Notably, the heat flow from the graphite–electrolyte reaction is lower in FMS than in DMC because the accelerated reaction between lithiated graphite and electrolyte is suppressed by continuous film formation at elevated temperatures. In FMS, the peak temperature increases by 13.5°C and the heat flow decreases by 29.2%. Thus, the thermal failure of graphite electrodes under abuse conditions can be mitigated by using FMS through SEI film‐composition modification and a reduction in the solvent volatility. The DSC‐based thermal stability analysis of the electrolyte–positive electrode interface in 50% delithiated NCM811 electrodes is shown in Figure 2b. Compared with the DMC system, the exothermic heat flow due to the electrolyte–positive electrode reaction is reduced by 74.7% in the FMS system [16], and the onset temperature of the exothermic reaction is increased by 23.9°C, indicating that the risk of thermal runaway due to positive electrode collapse is mitigated by the FMS electrolyte through the reinforcement of the interface with NCM. The accelerating rate calorimetry (ARC) testing results with 1.2‐A h graphite/NCM811 pouch cells charged to 70% SOC, used to compare the thermal stability of LIBs containing DMC and FMS, are shown in Figure 2c. The heat–wait–seek procedure is used to characterize the self‐heating of the pouch cells based on the previously discussed thermal failure mechanisms. In the DMC‐injected cell, self‐heating caused by SEI decomposition and electrode–electrolyte interface reactions is observed as a thermal plateau within 100°C–250°C [17, 21, 22], which ultimately results in thermal runaway at 180°C. In contrast, the FMS‐injected pouch cell shows no evidence of self‐heating at temperatures below 200°C, likely owing to significantly suppressed heat generation from SEI decomposition and electrode–electrolyte reactions. These calorimetry results are consistent with DSC and ARC measurements, further supporting cross‐validation. Specifically, the self‐heating onset temperatures of DMC were 115.1°C from DSC and 119.6°C from ARC, indicating reliability. Consequently, FMS effectively limits heat accumulation due to side reactions. An optical image of the pouch cell after ARC testing is shown in the inset of Figure 2c. As expected, the DMC‐injected pouch cell undergoes significant expansion owing to gas generation during thermal runaway, whereas the FMS‐injected cell retains its structural integrity on thermal cycling, indicating excellent thermal robustness.

Figure 2d,e shows the post‐mortem morphological analysis results of the positive and negative electrodes after ARC testing. The cross‐sectional scanning electron microscopy (SEM) image of the graphite electrode in the DMC system (Figure 2d) indicates interfacial failure at the graphite surface followed by electrode detachment from the current collector owing to gas evolution during thermal runaway. In contrast, the graphite electrode in the FMS system exhibits a well‐preserved structure, likely owing to the formation of a reinforced SEI film on the graphite surface and low heat generation due to the low volatility of the FMS electrolyte. Interestingly, the positive electrodes in both the DMC‐ and FMS‐injected pouch cells undergo similar morphological failure (Figure 2e). In the DMC electrolyte, the NCM811 active material undergoes detachment from the current collector owing to heat generation due to electrode–electrolyte reactions and gas evolution. In contrast, the electrode in FMS shows a well‐preserved structure, even on heating up to 200°C. The detachment of the active material from the current collector leads to partial mechanical contact between the negative and positive electrodes, which can cause short‐circuiting in LIBs. Therefore, the mitigation of morphological failure in both the positive and negative electrodes enhances the thermal stability of LIBs injected with the FMS electrolyte.

Figure 3 shows the thermal propagation test [52, 53] results with three‐stacked 1.2 A h pouch cells at 70% SOC with no additional inter‐cell pads, which were used to compare the electrolyte effect of DMC and FMS. A thermocouple is inserted between the cells, enabling surface‐temperature measurement using the TP test equipment shown. For the DMC‐injected cell, a temperature plateau at 270°C (which shows a marginal temperature increment owing to the non‐maintenance of an adiabatic state during TP testing) is observed at 2274 s, consistent with the results of ARC; subsequently, the thermal runaway increases abruptly, and a peak temperature is observed at 2515 s. In contrast, no notable temperature plateau is observed in the FMS electrolyte, likely due to the formation of a stable interface owing to the SO*_x_* component in the system. Furthermore, the peak temperature of the FMS system (at 3130 s) is significantly delayed (by 617 s) compared with that of the DMC system. This delay in thermal runaway time is unusual because the transformation of an FMS system to a DMC system involves only a change in electrolyte (not the separator). Furthermore, without the presence of typical inter‐cell pads, FMS application for 10 s mitigates thermal propagation from the first cell, which is in contact with the heating pad, to the last cell in the stacked‐cell system used for analysis.

**FIGURE 3:**
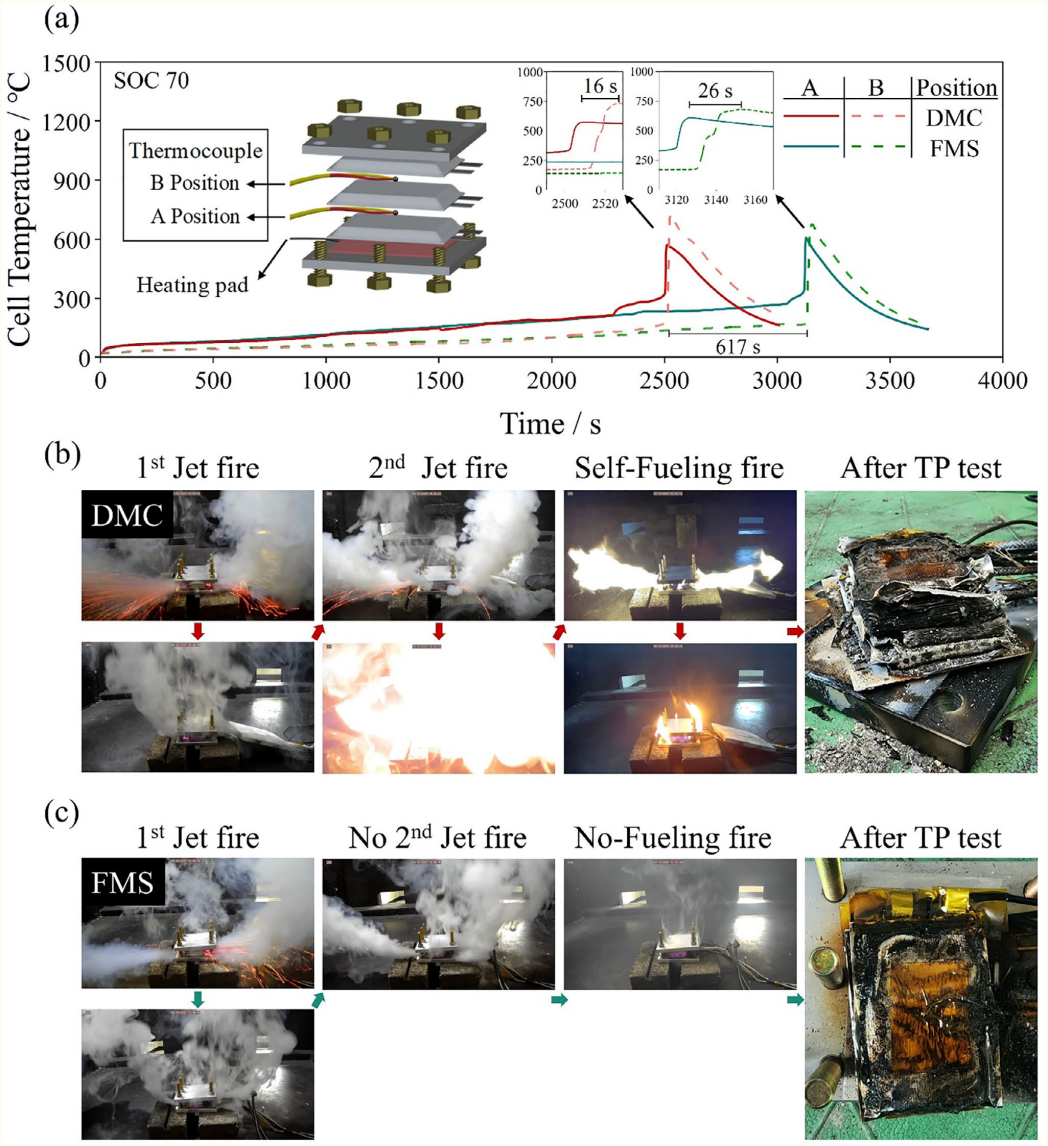
(a) Time vs. cell surface temperature profiles during thermal propagation testing (inset: schematics of the thermal propagation test setup). Time‐dependent optical images of cells injected with (b) DMC and (c) FMS during safety testing and after thermal propagation evaluation.

The cell images recorded during TP measurements with DMC and FMS are shown in Figure 3b,c. The thermal runaway process with a typical carbonate electrolyte involves a cascading failure with smoke generation, jet fire formation, and cell combustion. The DMC system exhibits typical thermal runaway behavior with cascading failure, culminating in the vigorous combustion of the LIB. In contrast, FMS largely suppresses jet‐fire formation during thermal runaway; therefore, the FMS system mitigates flame formation from LIB combustion through a stepwise mitigation mechanism involving the FMS electrolyte. This TP test was repeated to verify reproducibility. On heat application, the laminated pouch cells and stacked electrode structures in the DMC‐injected pouch cells undergo significant exfoliation, whereas the cell structure of the FMS‐based cells is preserved owing to the significant reduction of flame formation during heat application. Hence, the safety hazard due to opened pouch cells during thermal runaway can be prevented by applying the FMS electrolyte.

Figure 4 shows the electrochemical characterization of pouch cells injected with DMC and FMS. The cycling performance is evaluated at 45°C and charged 4.2 V because elevated‐temperature and wide voltage‐swing cycling under high energy loading and lean electrolyte conditions is typically used as an accelerated method for assessing the degradation of LIBs in electric vehicle (EV) and energy storage system (ESS) applications. The discharge energies from both the DMC and FMS electrolytes are similar because the increase in resistance due to FMS incorporation is only marginal, despite a significant improvement in thermal stability (Figure 4a). Furthermore, the discharge energy retention in FMS is higher than that in DMC because SO*_x_*‐based SEI and surface‐film formation on the graphite and NCM811 electrodes in FMS mitigate electrolyte decomposition, which typically increases the cell resistance during cycling.

**FIGURE 4:**
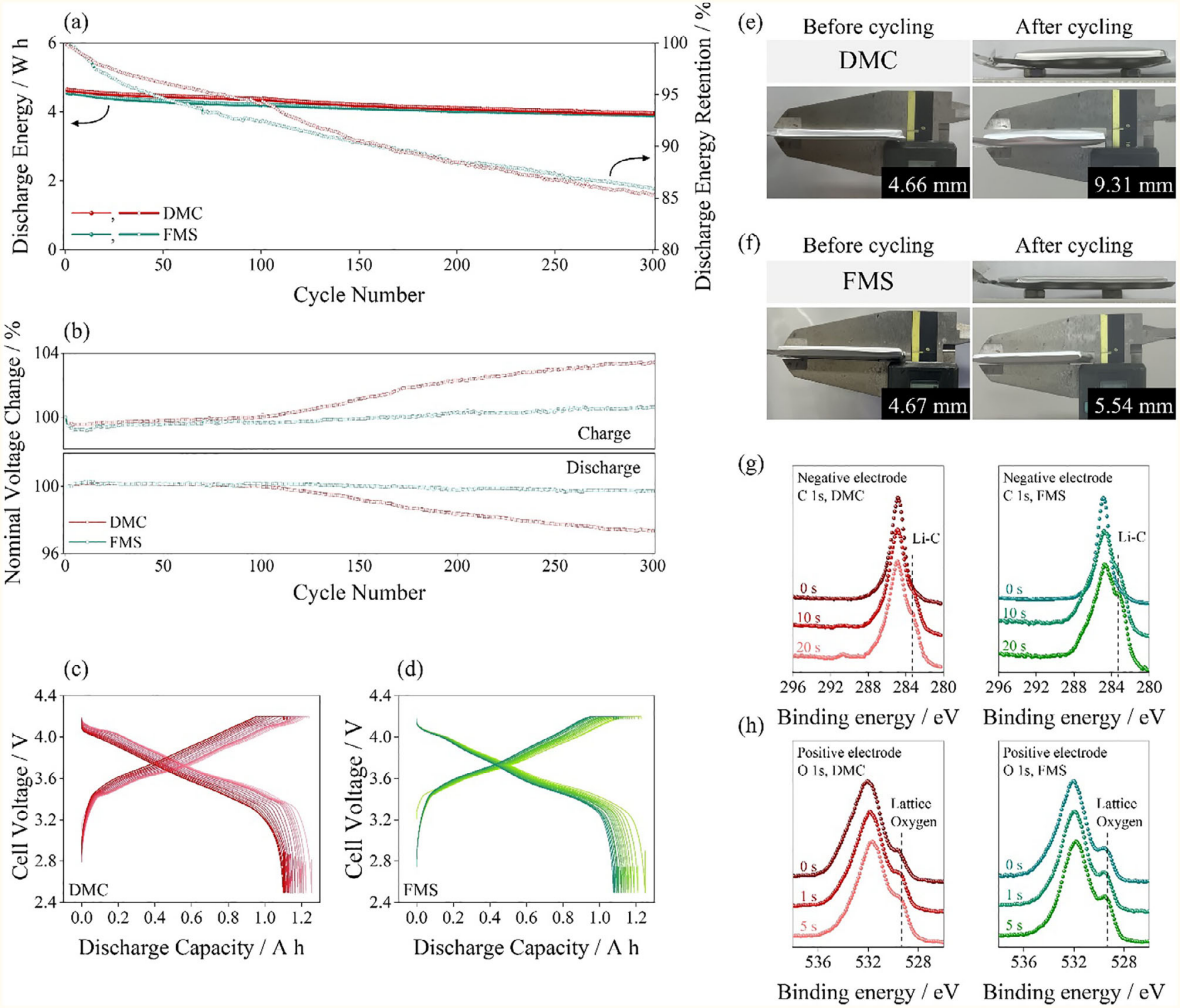
(a) Cell cycling performance at 45°C and 0.33 *C*, (b) nominal voltage changes during cycling, and cycle number‐dependent voltage profiles of the (c) DMC and (d) FMS‐injected pouch cells. Cell thickness and optical images of cycled cells with (e) DMC and (f) FMS electrolytes. Ex situ (g) C 1s XPS spectra of the negative electrode and (h) O 1s XPS spectra of the positive electrode cycled in DMC and FMS.

The nominal voltage change observed during cycling (Figure 4b) indicates that the voltage increases more sharply in DMC than in FMS, implying that the SO*_x_*‐based passivation film formed in FMS effectively suppresses electrolyte decomposition during elevated‐temperature cycling.

The cycle number‐dependent voltage profile of the DMC‐injected cell (Figure 4c) indicates polarization growth during elevated‐temperature cycling, which significantly decreases the discharge energy of LIBs; this is detrimental for EV and ESS applications because it involves a degradation of the battery power performance. In contrast, the FMS‐injected cell exhibits a well‐preserved voltage profile during high‐temperature cycling (Figure 4d), implying a well‐maintained power performance owing to the formation of an initially robust SEI film.

The thicknesses of the pouch cells after thermal cycling are shown in Figure 4e,f. After 300 cycles at 45°C, the thickness of the DMC‐based pouch cell increases by 99.8%, whereas that of the FMS‐injected pouch cell increases by only 18.6%, indicating marginal thickness growth in FMS.

These results confirm that the dimensional stability of the pouch cells is significantly improved in FMS owing to the suppression of side reactions by a robust passivation film derived from the FMS electrolyte.

In Figure 4g,h, the thicknesses of the SEI film on graphite and the surface film on NCM after cycling are compared using post‐mortem XPS spectra. The C 1s narrow‐scanned spectrum of the graphite electrode cycled in FMS contains a more prominent Li─C peak than the spectrum of the same electrode cycled in DMC [54, 55], indicating significant SEI film growth during high‐temperature cycling in DMC owing to the insufficient passivation ability of the electrolyte. Moreover, the O 1s XPS spectrum of the positive electrode cycled in FMS contains a more prominent lattice oxygen peak [43, 56, 57] from NCM than the spectrum of the same electrode cycled in DMC, indicating that the deposition of a highly passivating film in FMS suppresses further film deposition on the NCM surface. Because the growth of the interfacial resistance due to further electrolyte decomposition is mitigated by FMS, a nominal voltage change occurs in the system. Therefore, compared with conventional carbonates, FMS significantly improves the discharge energy retention in LIBs.

Figure S9 shows the SET test results for FMS mixed with conventional linear carbonate solvents. The electrolytes containing more than 20 vol.% FMS in DMC exhibited nonflammable behavior, confirming the possibility of FMS and DMC as a co‐solvent electrolyte system. As shown in Figure S10, the rate capability and discharge energy of the FMS electrolyte are optimized by mixing it with conventional linear carbonates. Introducing 50 vol.% of FMS into the DMC electrolyte (by substituting DMC) maintains the non‐flammability of the electrolyte (Figure S10a) while improving its discharge energy (Figure S10b); the rate capability (Figure S10c) of the modified system remains similar to that of the conventional DMC‐only electrolyte, with no capacity degradation observed during recovery. The mixed electrolyte effectively suppressed electrolyte decomposition and polarization growth through SO*_x_*‐based SEI film formation, confirming that FMS functions effectively both as a co‐solvent and as an additive. Therefore, the FMS content of DMC electrolytes can be strategically formulated to selectively modulate the safety and power performance of LIB systems. Notably, cells show significantly better cycling performance in FMS than in other nonflammable electrolytes reported to date, even at elevated temperatures (Table S1), and the LiPF_6–_FEC–FMS electrolyte system improves the safety of LIBs, including thermal propagation, in an effective manner.

Figure 5 shows the stepwise working mechanism of the FMS and DMC electrolytes under abusive conditions. During the formation period, the FMS electrolyte facilitates the development of thermally stable SO*_x_*‐based passivation films on the graphite and NCM811 electrode surfaces. These stable interfacial layers mitigate the thermal dissolution and decomposition of the passivation film, suppressing the decomposition and repeated formation of the SEI at elevated temperatures, which are both exothermic reactions. This SEI reformation process is commonly accompanied by the rapid self‐discharge of the graphite electrode, which leads to Joule heating in LIBs. Therefore, the introduction of FMS into the electrolyte effectively reduces heat accumulation within the cell. In contrast, heat accumulation owing to interfacial failure causes electrolyte vaporization in DMC, which increases the risk of ignition and leads to additional thermal failure. An increase in cell temperature can be induced by this thermal failure, resulting in thermal runaway in the LIB. Notably, the formation of a robust passivation layer ensures minimal heat accumulation in FMS, which exhibits a higher vaporization temperature than DMC owing to an asymmetric molecular structure, resulting in the suppression of ignition and subsequent thermal runaway in LIBs. Even when the temperature increases beyond the point of interfacial failure, direct reactions between the exposed electrode and electrolyte are suppressed in the FMS system. Thus, owing to the presence of a durable interfacial film that inhibits extensive side reactions, limited heat is released during these reactions in FMS. This progressive suppression of thermal runaway significantly improves battery safety, as confirmed by ARC and thermal propagation tests. Additionally, the use of an FMS electrolyte enhances the cycling performance and dimensional stability of pouch cells by reducing undesired side reactions.

**FIGURE 5:**
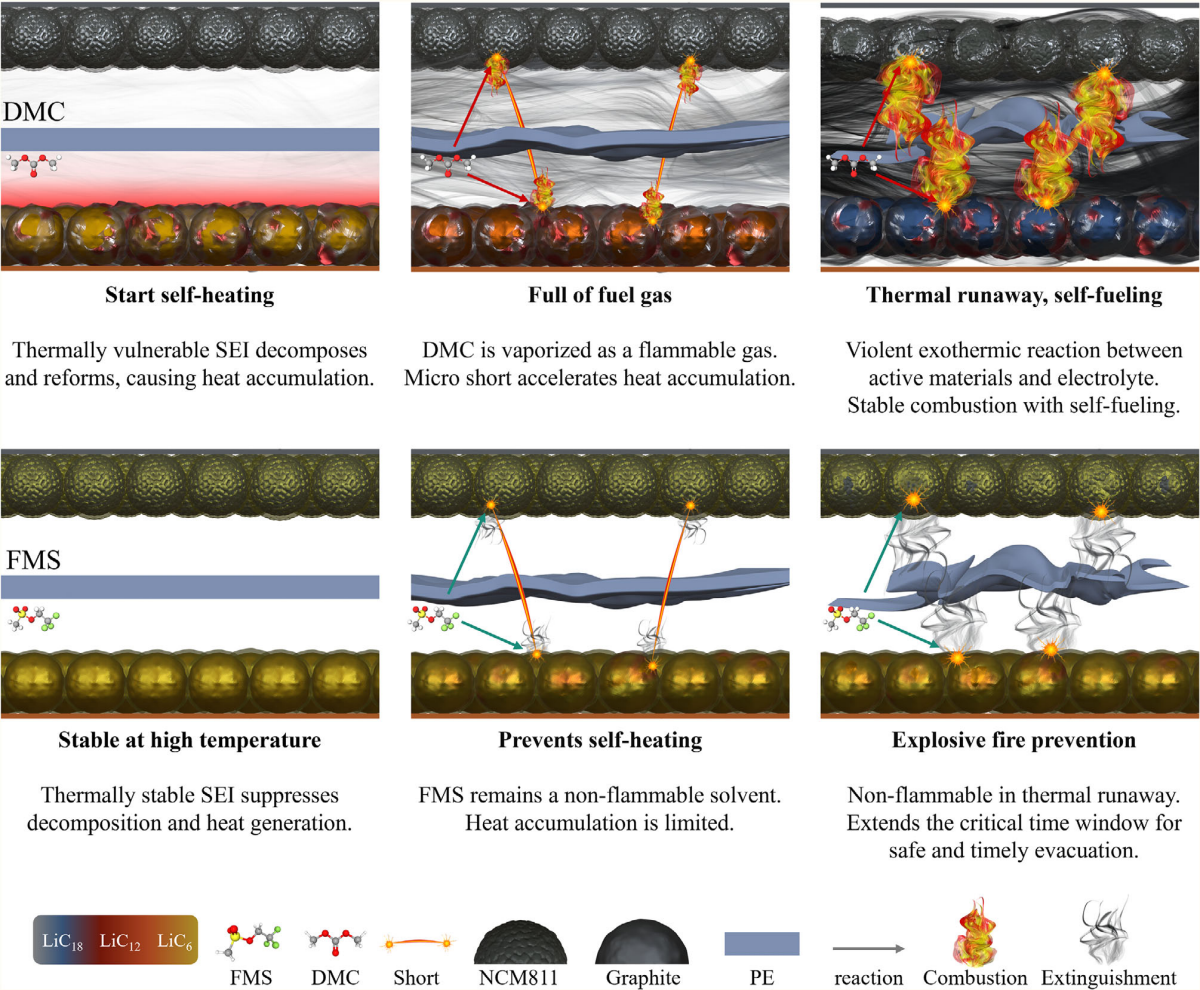
Schematics of the working mechanism of the DMC and FMS electrolytes.

## 3 Conclusion

In summary, the results of this study confirm the potential utility of novel electrolyte solvents containing FMS in enhancing the thermal safety and electrochemical stability of LIBs under harsh conditions. FMS‐based solvents enable the formation of highly stable SO*_x_*‐based passivation layers on both the graphite and NCM811 electrode surfaces in LIBs, resulting in the suppression of thermal decomposition and SEI formation, which typically lead to heat accumulation from self‐discharge. Consequently, the overall heat generation within cells is significantly reduced. Moreover, the asymmetric molecular structure of FMS enables FMS‐containing electrolytes to exhibit a higher vaporization temperature than conventional solvents, effectively minimizing the risks of ignition and thermal runaway in LIBs. Even at elevated temperatures, where interfacial breakdown normally leads to severe side reactions, the presence of a resilient passivation film limits further electrode–electrolyte reactions in FMS systems, controlling the heat release from these exothermic reactions. The stepwise suppression of thermal failure mechanisms by FMS improves the safety of LIBs, even in thermal propagation tests. Additionally, owing to the suppression of side reactions by a robust surface film, pouch‐type cells show good cycle performance and dimensional stability in FMS, confirming the practical benefits of the proposed FMS‐based solvent design. The results of this study are expected to open new frontiers in LIB research by promoting the design and development of high‐performance systems with significant potential for practical application.

## 4 Experimental Section

### 4.1 Electrolyte Formulation and Physicochemical Characterization

An Ar‐filled glove box (Siemens) was used for electrolyte formulation. Before use, FMS (TCI Chemicals) was pre‐treated with activated molecular sieves (4 Å, 4–8 mesh, Samchun Chemical) to control moisture contaminants. Three types of electrolytes were prepared: a conventional carbonate electrolyte synthesized by dissolving LiPF_6_ (1.0 M; Chunbo) in DMC (Soulbrain) and FEC (EnChem) in a 9:1 volume ratio, a nonflammable electrolyte consisting of LiPF_6_ (1.0 *M*) dissolved in a mixture of FMS and FEC in a 9:1 volume ratio, and a mixed‐solvent electrolyte comprising LiPF_6_ (1.0 *M*) in a mixture of FMS, DMC, and FEC in a 5:4:1 volume ratio. During electrolyte synthesis, the Li salt was dissolved in the relevant solvents by shaking for 12 h in a shaker (Daihan Scientific).

### 4.2 Pouch Stack‐Cell Fabrication and Formation

Positive electrodes were fabricated using a mixture of the active material NCM811 (BTR), conductive material Super P (Timcal), and binder polyvinylidene fluoride (Kureha, KF‐1100) dispersed in *N*‐methyl‐2‐pyrrolidone (NMP, Sigma–Aldrich) in a 96:2:2 weight percentage (wt.%) ratio. The resulting slurry was cast onto an aluminum foil and pre‐dried at 80°C before drying under vacuum for 12 h at 120°C. To fabricate the negative electrodes, a water‐based slurry consisting of the active material graphite (BTR), conductive material Super P, and a binder comprising styrene‐butadiene rubber (SBR; Sigma–Aldrich) and carboxymethyl cellulose (CMC; Sigma–Aldrich) (SBR:CMC = 1:1 wt.% ratio) mixed in a 90:5:5 wt.% ratio was cast onto copper foil. The coated electrodes were pre‐dried at 80°C before drying under vacuum for 12 h at 80°C. Pouch cells with a discharge capacity of 1.2 A h and negative/positive electrode capacity ratio (N/P ratio) of 1.08 were assembled with a positive electrode, PE separator, and negative electrode. Each pouch cell was injected with the relevant electrolyte (3.0 g; DMC, FMS, or MIX) at an electrolyte‐to‐capacity (E/C) ratio of 2.5 g (A h)^−1^, degassed (Topre), and stored at room temperature for 24 h to ensure electrolyte wettability. The cell formation process (TOYO Japan) was conducted under 1.0 MPa pressure using a jig. The cell performance was monitored under a constant current (CC) charge of 0.1 *C* at 4.2 V with a constant voltage (CV) hold until the current reached 0.05 *C*, and under CC discharge at 0.1 *C* and 2.5 V. A 0.33 *C* CC–CV charge (0.05 *C* cut‐off) and 0.33 *C* CC discharge in the voltage range of 2.5–4.2 V at 25°C were used for the grading process. For the cycle performance tests, a 0.33 *C* CC–CV (0.05 *C* cut‐off) charge and 0.33 *C* discharge in the voltage range of 2.5–4.2 V at 45°C were used.

### 4.3 Density Functional Theory Calculation Details

The HOMO and LUMO energy levels and Li‐ion binding energy were computed using geometry optimization at the B3LYP functional with a triple‐zeta valence polarization basis set within the ORCA software suite (version 4.2.1) [1, 2, 3, 4, 5, 6, 7, 8, 9, 10, 11]. The Li‐ion binding energy was determined using the following equation:

$$Li-ion\;binding\;energy=E_{Li-ion}+E_{anion}-E_{Ionic\;compound}$$

### 4.4 Self‐Extinguishing Time Tests of Electrolytes With a Separator

PE separators (Toray) cut to a width of 1.0 cm were placed in glass crucibles and soaked in each electrolyte (180 µL). A torch flame (butane gas) was applied to each soaked sample for 2.0 s, and the self‐extinguishing time (until the fire was extinguished) was recorded. Snapshots were recorded every 4.0 s, and the time of the last snapshot was recorded as the self‐extinguishing time. In cases where ignition was not observed, a re‐ignition test was conducted under the same conditions as the initial test to confirm the non‐flammability of the solvent.

### 4.5 Contact Angle Tests

Contact angles were measured using a contact angle measurement instrument (Kruss Optronic) to evaluate the wettability between the electrolyte (DMC or FMS) and the positive electrode (NCM811, 3.2 g cc^−1^) surface. During analysis, a droplet (2.0 µL) of each electrolyte was placed onto the electrode surface at room temperature.

### 4.6 *e* *x*‐*s* *itu* Spectroscopic and Morphological Analyses

Spectroscopic and morphological analyses of the electrodes cycled in DMC and FMS were conducted in a controlled dry‐room environment using XPS (Thermo Fisher Scientific), SEM (JEOL), ToF‐SIMS (ION‐ToF), and cryogenic electron microscopy (*cryo*‐TEM, Mel‐Build). Prior to analysis, the electrodes were rinsed with DMC or FMS to remove the residual electrolyte and salt and dried under vacuum for 12 h. Further, to investigate the cross‐sectional SEM images of electrode structures, the specimens were polished using a cryogenic cross‐sectional polisher (*cryo*‐CP, JEOL) at −50°C.

### 4.7 Graphite Half‐Cell Self‐Discharge Tests at the Negative Electrode

A graphite electrode, identical in material composition to the negative electrode of the pouch cell, was fabricated with a mass loading of 2.4 mg cm^−2^. Subsequently, graphite half‐cells were prepared using the graphite electrode as the working electrode, Li metal foil (200 µm, Honjo Metal) as the counter and reference electrode, and a PE separator. The electrolyte (60 µL; DMC or FMS) was then injected into the 2032 coin cell. The formation process (Maccor) involved three formation cycles of 0.1 *C* CC–CV discharge (0.05 *C* cut‐off) and 0.1 *C* CC discharge within 0.001–1.5 V at 25°C. After formation, the discharged cells were stored at 60°C for 7 days while monitoring the OCV.

### 4.8 Differential Scanning Calorimetry Analysis Protocol

After the first formation cycle, the positive electrodes extracted from the pouch cells were punched into samples with a diameter of 4.0 mm without rinsing or drying to preserve the residual electrolytes. The electrodes were then sealed in aluminum pans (Mettler Toledo) and used for thermal analysis under an inert argon atmosphere; during analysis, the samples were heated from 25°C to 400°C at a rate of 2.5°C min^−1^ to evaluate exothermic and endothermic reactions by DSC (Mettler Toledo). To prepare SOC 100 negative electrodes, the negative and positive electrodes of the pouch cell were punched to diameters of 14 and 12 mm, respectively. Each electrolyte was used to assemble a 2032 coin cell. These cells were charged with 0.1 *C* CC–CV (0.05 *C* cut‐off) to 4.2 V. Identical to the DSC sampling method used for the positive electrodes, the lithiated graphite electrodes obtained from the cells were punched to a diameter of 4.0 mm before analysis. During DSC, the temperature was increased from 25°C to 350°C at a temperature ramp rate of 2.5°C min^−1^. The DSC results were calculated based on the weights of the electrodes.

### 4.9 ARC Protocol and Post‐Analysis

Following the formation process, the 1.2 A h pouch cells were charged to SOC 70 using a 0.1 *C* CC protocol. ARC (THT) was performed using the heat–wait–seek method to determine the runaway temperatures of the cells. The measurements were conducted in a THT EVx calorimeter chamber (25 cm (D) x 60 cm (H)) with a temperature sensitivity of 0.02°C min^−1^ over a temperature range of 40–350°C at a heating rate of 2.0°C min^−1^. After ARC analysis, the thickness of the pouch cells was measured using a digital caliper (Mitutoyo). The cross‐sections of the disassembled electrodes were polished using *cryo*‐CP and examined using SEM. To protect the SEI layer from electron beam damage, a liquid nitrogen TEM holder (Mel‐Build) was utilized. Following specimen loading into the TEM, the sample was further cooled to −160°C. The TEM observations were subsequently conducted at 200 kV accelerating voltage using a JEOL JEM‐ARM 200F microscope (JEOL). Energy‐dispersive x‐ray spectroscopy (EDS) spectra and elemental maps were collected using an X‐Max 100TLE detector (Oxford Instruments).

### 4.10 Thermal Propagation Tests

Following the formation process, three 1.2 A h pouch cells comprising the DMC‐ and FMS‐based systems were charged to SOC 70 using the 0.1 *C* CC protocol. For thermal propagation testing, insulation tape was applied to the lead tap of each cell to cut off the electrical connections between the cells. The following order of stacking was used in the tests: the heating pad, lower cell, first thermocouple, middle cell, second thermocouple, and top cell. The heating pad was heated at a rate of 5°C min^−1^ to induce thermal runaway by supplying heat to the lower cell. A thermocouple was used to measure the heat propagating through the cells, and pressure was applied using a jig to maintain the stacked structure. These tests were conducted under atmospheric conditions, and the cell temperature was measured using a thermocouple. The repetition process was recorded using a camera. After thermal propagation testing, the jig was disassembled, and optical images of the cells were recorded.

### 4.11 Rate Performance Evaluation

Following cell formation, their rate performance was assessed at 25°C using a fixed charge rate of 0.33 *C* CC–CV (0.05 C cut‐off), sequential rates of 0.33, 0.66, 1.0, 1.5, and 2.0 *C* for CC discharge, and finally 0.33 *C* CC to evaluate the reversible capacity retention. The thickness of the pouch cells after ARC analysis was measured using a digital caliper.

### 4.12 Computational Details

The dipole moments and ESP maps of the molecules (DMC, FMS and FEC) were calculated using the Q‐Chem 5.4.0 package. Molecular geometries were optimized at the B3LYP/6‐311++G(d,p) level of theory [58, 59]. Frequency calculations confirmed that all optimized structures were true global minima with no imaginary frequencies.

Intermolecular interaction energies (molecule–molecule interactions, molecule—Li^+^ ion binding) and ab initio molecular dynamics (AIMD) simulations were performed using the Vienna Ab Initio Simulation Package (VASP, version 5.4.4) [60, 61]. These calculations employed the projector augmented‐wave (PAW) method and Perdew–Burke–Ernzerhof (PBE) exchange‐correlation functional with Grimme's D3 dispersion correction [62, 63, 64]. A kinetic cutoff energy of 400 eV was used, and the convergence criteria for energy and force were set to 10^−4^ eV and 0.05 eV/Å, respectively. The gamma‐centered mesh grid was used to sample the Brillouin zone [65]. The binding energy (∆E_b,_ *_i_*) of molecule *i* (*i* = DMC, FMS, FEC) to solvation structure (SS) was calculated as follows [1]:

$$\Delta \;E_{b,i}=E_{SS}\;-E_{SS-i}-E_{i}$$

where *E_SS_*, *E_SS‐i,_* and *E_i_* are the DFT‐calculated energies of the optimized total solvation structure, the solvation structure without molecule *i*, and the isolated molecule *i*, respectively. All calculations were performed within a 20 Å × 20 Å × 20 Å unit cell. For the AIMD simulations, two electrolyte systems were modeled: one based on DMC and the other on FMS. The compositions were 1 Li^+^ + 1 PF_6_^−^ + 21 DMC + 3 FEC for the DMC system, and 1 Li^+^ + 1 PF_6_^−^ + 15 FMS + 2 FEC for the FMS system. To investigate the solvation structure formed by electrolyte molecules, the dissociated configuration of Li^+^ + PF_6_^−^ was used as the initial structure. The simulation boxes were adjusted to match densities of 1.26 g/cm^3^ (DMC) and 1.62 g/cm^3^ (FMS), corresponding to a LiPF_6_ salt concentration of 0.53 M. Although experimental conditions typically involve 1 M LiPF_6_, we employed a lower concentration of 0.53 M to minimize inter‐ionic effects and isolate the behavior of a single solvation structure, while still reproducing the experimental density [1, 66]. The AIMD simulations of electrolyte structures at 330 K were performed in the NVT ensemble, with the temperature controlled by a Nosé–Hoover thermostat [67]. Each system was equilibrated for 10 ps, followed by a 10 ps production run for statistical sampling. The interaction energy between the primary solvation structures and the surrounding electrolyte was calculated using the following equation:

$$\Delta \;E_{SS}=E_{Total}^{sc}\;-E_{SS}^{sc}-E_{Surr}^{sc}$$

Here, all energies were obtained from single‐point DFT calculations on atomic configurations extracted from AIMD trajectories. Specifically, $E_{Total}^{sc}$, $E_{SS}^{sc}$ and $E_{Surr}^{sc}$ represent the DFT energies of the entire electrolyte structure, the isolated primary solvation structure, and the surrounding electrolyte, respectively.

## Author Contributions

**Ho Yeon Jang**: methodology, software, investigation, visualization. **Jeongmin Kim**: methodology. **Chihyun Hwang**: formal analysis, writing – review and editing, investigation, validation. **Jooeun Byun**: methodology, validation, formal analysis. **Oh B. Chae**: methodology. **Jun Ho Song**: resources, supervision, formal analysis. **Joon Ha Chang**: methodology, investigation, visualization, validation, formal analysis. **Youngjin Kim**: writing – review and editing, validation, formal analysis. **Chae Rim Lee**: methodology, validation, formal analysis. **Chi‐Yeong Hong**: writing – original draft, methodology, data curation, formal analysis, validation, investigation. **Hyun‐seung Kim**: conceptualization, software, data curation, formal analysis, validation, investigation, funding acquisition, visualization, writing – original draft, writing – review and editing, project administration, resources, supervision. **Ki Jae Kim**: writing – review and editing, methodology, validation. **Seoin Back**: writing – review and editing, investigation, software, validation, visualization.

## Funding

This work was supported by the Ministry of Trade, Industry & Energy/Korea Evaluation Institute of Industrial Technology (MOTIE/KEIT, No. RS‐2026‐25535290) and the Competitiveness reinforcement project for industrial clusters (HUKB2305) supervised by the Ministry of Trade, Industry and Energy (MOTIE). S.B. acknowledges the support from the National Research Foundation of Korea (NRF) grant funded by the Korea government (MSIT) (RS‐2025‐02214715)

## Conflicts of Interest

The authors declare no conflicts of interest.

## Supporting information




**Supporting File**: advs77307‐sup‐0001‐SuppMat.docx.

## Data Availability

The authors have nothing to report.
