# Supplementary material for: Flame Suppression in Lithium‐Ion Batteries During Thermal Runaway Through Solvent Design Considering the Volatility–Flammability Relationship
Source: Adv Sci (Weinh). 2026 Aug 19:e77307. Online ahead of print. doi: 10.1002/advs.77307 (PMC13487865; doi:10.1002/advs.77307)
Supplement: Supplementary file 1 [file ADVS-9999-e77307-s001.docx]

Supporting Information

**Flame Suppression in Lithium-Ion Batteries during Thermal Runaway through Solvent Design Considering the Volatility–Flammability Relationship**

*Chi-Yeong Hong, Joon Ha Chang, Ho Yeon Jang, Jooeun Byun, Chae Rim Lee, Jeongmin Kim, Oh B. Chae, Youngjin Kim, Jun Ho Song, Chihyun Hwang*, Seoin Back^*^ , Ki Jae Kim^*^
and Hyun-seung Kim^*^*

**Figure S1**. The binding energy values (∆E*_b,i_*) of solvent molecules (DMC, FMS, FEC). The hatched bars indicate the binding energy for the FEC molecule. Color codes: Purple (Li), orange (P), green (F), red (O), white (H), gray (C), yellow (S).

**Figure S2**. The initial and final structure, along with the energy profiles from AIMD simulations for (a) the DMC system and (b) the FMS system. Color codes: Purple (Li), orange (P), green (F), red (O), white (H), gray (C), yellow (S).

Figure S3. Measured contact angle of DMC and FMS electrolyte at NCM811 positive electrode

**Figure S4**. top-most (a) C 1s, (b) S 2p, (c) O 1s, (d) F 1s XPS spectra obtained from negative electrodes after formation with DMC and FMS electrolytes, respectively

**Figure S5**. top-most (a) O 1s, (b) S 2p, (c) C 1s, (d) F 1s XPS spectra obtained from positive electrodes after formation with DMC and FMS electrolytes, respectively

**Figure S6**. (a), (b) XPS depth profile obtained from negative electrodes after formation with DMC and FMS electrolytes, respectively.

**Figure S7**. EDS spectrum corresponding to EDS map in Figure 1f. (inset: magnified spectra at the range of 0.4-2.5 keV energy)

**Figure S8**. (a) storage time dependent OCVs of Li/graphite cell at SOC 0 with DMC and FMS electrolytes, (b) Li-ion binding energy of Li_2_SO_3_, lithium ethyl decarbonate, and lithium methyl carbonate

**Figure S9**. Flammability test according to the DMC : FMS : FEC ratio

**Figure S10**. (a) flammability test, (b) cycle performance (inset: cycle number-dependent voltage profiles), and (c) rate capability test with FMS-partially substituted electrolyte

**Table S1**. Comparison of FMS electrolyte with reported result

**References**

[1]	Chen, L., Shen, X., Chen, H., Wen, T., Rao, R., Zhang, C., ... & Chen, Z. (2023). High-stable nonflammable electrolyte regulated by coordination-number rule for all-climate and safer lithium-ion batteries. Energy Storage Materials, 55, 836-846, [10.1016/j.ensm.2022.12.044](https://doi.org/10.1016/j.ensm.2022.12.044)

[2]	Lu, D., Zhang, S., Li, J., Huang, L., Zhang, X., Xie, B., ... & Cui, G. (2023). Transformed Solvation Structure of Noncoordinating Flame‐Retardant Assisted Propylene Carbonate Enabling High Voltage Li‐Ion Batteries with High Safety and Long Cyclability. Advanced Energy Materials, 13(28), 2300684, [10.1002/aenm.202300684](https://doi.org/10.1002/aenm.202300684)

[3]	An, K., Tran, Y. H. T., Kwak, S., Han, J., & Song, S. W. (2021). Design of fire‐resistant liquid electrolyte formulation for safe and long‐cycled lithium‐ion batteries. Advanced Functional Materials, 31(48), 2106102, [10.1002/adfm.202106102](https://doi.org/10.1002/adfm.202106102)

[4]	Wen, F., Cao, S., Ren, X., Cao, Y., Ai, X., & Xu, F. (2022). Nonflammable dual-salt electrolytes for graphite/LiNi0. 8Co0. 1Mn0. 1O2 lithium-ion batteries: Li+ solvation structure and electrode/eelectrolyte interphase. *ACS Applied Energy Materials*, *5*(12), 15491-15501, [10.1021/acsaem.2c03065](https://pubs.acs.org/doi/abs/10.1021/acsaem.2c03065)
